# Initial orthostatic hypotension and orthostatic intolerance symptom prevalence in older adults: A systematic review

**DOI:** 10.1016/j.ijchy.2020.100071

**Published:** 2020-12-08

**Authors:** Elena M. Christopoulos, Jennifer Tran, Sarah L. Hillebrand, Peter W. Lange, Rebecca K. Iseli, Carel G.M. Meskers, Andrea B. Maier

**Affiliations:** a Department of Medicine and Aged Care, @AgeMelbourne, The Royal Melbourne Hospital, University of Melbourne, Melbourne, Australia; b Department of Human Movement Sciences, @AgeAmsterdam, Faculty of Behavioural and Movement Sciences, VU University Amsterdam, Amsterdam Movement Sciences, Amsterdam, the Netherlands; c Department of Rehabilitation Medicine, Amsterdam UMC, VU University Medical Center, Amsterdam Movement Sciences, Amsterdam, the Netherlands

**Keywords:** Hypotension, Orthostatic intolerance, Symptoms, Blood pressure, Syncope, Frail elderly, IOH, initial orthostatic hypotension, COH, classical orthostatic hypotension, BP, blood pressure, SBP, systolic blood pressure, DBP, diastolic blood pressure

## Abstract

**Background:**

Initial orthostatic hypotension is a clinically relevant syndrome in older adults which has been associated with symptoms of orthostatic intolerance. The aim of this systematic review was to determine the prevalence of orthostatic intolerance symptoms in older adults with initial orthostatic hypotension.

**Methods:**

MEDLINE (from 1946), EMBASE (from 1974) and Cochrane were searched to December 6th^,^ 2019 using the terms “initial orthostatic hypotension”, “postural hypotension” and “older adults”. Study selection involved the following criteria: published in English; mean or median age ≥65 years and diagnosis of initial orthostatic hypotension encompassed a decrease in systolic blood pressure by ≥ 40  mmHg and/or diastolic blood pressure by ≥ 20  mmHg within a maximum of 1 min following a postural change.

**Results:**

Of 8311 articles, 12 articles reporting initial orthostatic hypotension prevalence in 3446 participants with a mean age of 75 (6 SD) years (56.5% female) were included. Five initial orthostatic hypotension definition variations were utilised and symptoms were reported in six articles (968 participants, mean age 73.4 (6.1 SD) years, 56% female). The prevalence of symptoms in older adults with initial orthostatic hypotension ranged from 24 to 100% and was dependent on variations in timing or the inclusion of symptoms in the initial orthostatic hypotension definition.

**Conclusions:**

Where orthostatic intolerance symptoms were reported, a large proportion of older adults with a diagnosis of initial orthostatic hypotension were symptomatic. However, the literature on initial orthostatic hypotension and orthostatic intolerance symptoms is scarce and a variety of definitions of initial orthostatic hypotension are utilised.

## Highlights

• Symptoms of orthostatic intolerance occur with initial orthostatic hypotension.
• 24–100% of older adults with initial orthostatic hypotension experienced symptoms.
• Symptom prevalence is affected by varying initial orthostatic hypotension definitions.

## 1 Introduction

Initial orthostatic hypotension (IOH) is classified by a transient drop in systolic blood pressure (SBP) by ≥40 mmHg and/or diastolic blood pressure (DBP) by ≥20 mmHg within 15 s of a change in posture from a supine to an upright position [1]. IOH is most accurately detected by continuous blood pressure monitoring [2] and ranges in prevalence from 3.5% [2] to 62.5% [3] in geriatric outpatients aged 65 years and older. Both IOH and classical orthostatic hypotension (COH); a drop in SBP by ≥20 mmHg and/or a drop in DBP by ≥10 mmHg within 3 min of a postural change [1], are two of several syndromes of orthostatic intolerance that often though not always provoke orthostatic intolerance symptoms [4]. Several variations of the consensus IOH definition are utilised, which vary in the timing of the blood pressure drop and whether or not the blood pressure drop coincides with the experience of symptoms of orthostatic intolerance [5,6].

Orthostatic intolerance symptoms are caused by transient cerebral hypoperfusion [7,8] and typically occur upon standing during the designated time period for the drop in blood pressure and resolve upon returning to baseline blood pressure or recumbency. Orthostatic intolerance most commonly presents as presyncope symptoms including dizziness, light-headedness and instability or weakness upon standing [9], but can also encompass symptoms such as headache, blurred vision, nausea, palpitations, chest pain and syncope upon standing [10]. Orthostatic intolerance symptoms in older adults with COH have been associated with decreased quality of life [11,12], higher risks of falls, syncope, fractures [13], morbidity and mortality [12,14]. The prevalence of symptoms of orthostatic intolerance in older adults diagnosed with COH ranges from 20% to 33% [15,16], however, evidence of the association between IOH and orthostatic intolerance symptoms in older adults is limited and inconclusive [2,[17], [18], [19]].

The aim of this systematic review was to determine the prevalence of orthostatic symptoms in older adults with IOH aged 65 years or older.

## 2 Methods

### 2.1 Search strategy

The systematic review protocol was registered with PROSPERO International prospective register of systematic reviews (registration number: CRD42020170696) [20] and was produced in accordance with the Preferred Reporting Items for Systematic Reviews and Meta-Analyses (PRISMA) guidelines [21]. The databases Ovid MEDLINE (from 1946), EMBASE (from 1974) and Cochrane Central Register of Controlled trials were searched from inception until December 6th^,^ 2019. The search strategy involved key words including “initial orthostatic hypotension”, “postural hypotension” and “older adults” and is displayed in Supplementary Material 1.

### 2.2 Article selection

All identified articles were managed with the referencing management software EndNote (Version: X9 Clarivate Analytics, Philadelphia, USA). After elimination of duplicates, the remaining articles were exported into Covidence systematic review software (Veritas Health Innovation, Melbourne, Australia) and were assessed by screening the titles and abstracts by two independent reviewers (JT and SLH). The full texts of articles were screened by the same two reviewers to obtain the final included articles. Any disagreements between the two reviewers were resolved by a third reviewer (RKI).

### 2.3 Eligibility criteria and data extraction

Articles were eligible for inclusion in this systematic review if they met the following inclusion criteria: 1) mean or median age of 65 years or older of the (subgroup of the) cohort, 2) all study designs or settings, 3) IOH diagnosis was defined as a decrease in SBP by ≥ 40  mmHg and/or DBP by ≥ 20  mmHg within a maximum duration of 1 min post postural change, 4) articles were published in English. The exclusion criteria included: 1) IOH induced experimentally by the use of medication, 2) case reports of less than five participants, reviews, conference abstracts, editorials and letters to the editor. The reference lists from all included full-text articles were examined to identify any potential additional articles. Two reviewers (EC, JT or SLH) extracted the following variables: first author, publication year, population studied, priori selection criteria, study design, study setting, number of participants, percentage of valid blood pressure measurements, percentage of females, mean or median age, IOH and COH definition, prevalence of IOH and COH, resting period (min), standing period (min), type of postural change (active stand, passive stand, supine-sit or passive supine-sit), type of blood pressure measurement (continuous or intermittent), type of device utilised to measure blood pressure, prevalence of orthostatic intolerance symptoms and orthostatic intolerance symptom prevalence dependent on IOH and COH diagnosis. Active stand was defined as standing autonomously, passive stand was defined as the immobility or inactivation of lower limb muscles during the transition from supine to standing. Supine-sit was defined as actively moving from supine to an upright sitting position and passive supine-sit was defined as inactivation of abdominal and limb muscles while being moved to an upright sitting position.

### 2.4 Study quality and risk of bias assessment

The quality and risk of bias of each included article was assessed by two independent reviewers (EC, JT or SLH) using the nine-point Newcastle-Ottawa Scale (NOS) [22] for cross-sectional studies demonstrated in Supplementary Material 2, by assigning each included article a score between 0 and 9. Articles with a NOS score between 0 and 3 points were classified as low quality, 4–6 points as moderate quality and 7–9 points as high quality [22].

## 3 Results

### 3.1 Study selection and search strategy

**Fig. 1:**
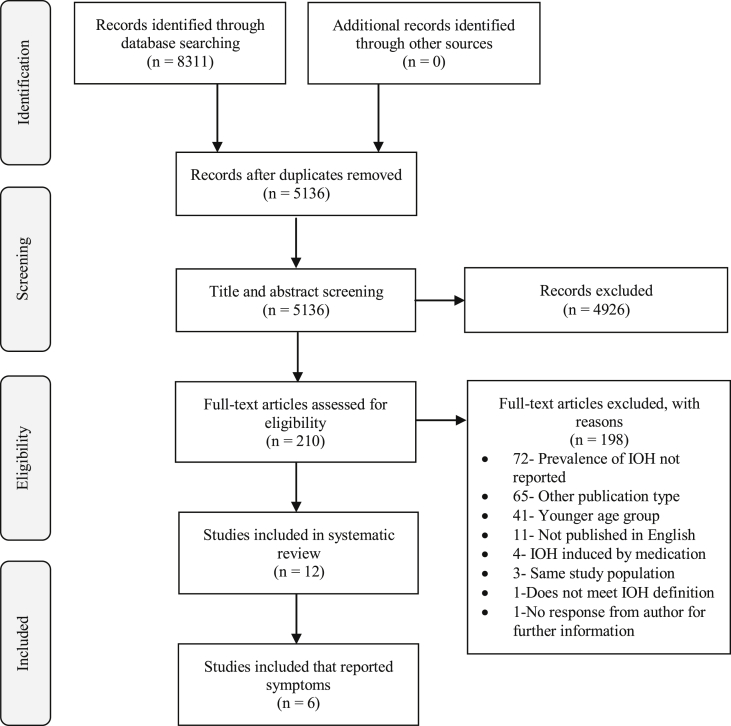
Flow diagram of the selected studies for the systematic review according to the Preferred Reporting Items for Systematic reviews and Meta-Analyses (PRISMA). IOH: initial orthostatic hypotension.

The process of identification, screening and selection of included articles is illustrated in the PRISMA flow diagram in Fig. 1. Out of 8311 articles, 5136 remained after removal of duplicates that were screened by title and abstracts. Of the 210 articles that underwent full text screening, 12 articles were included in the systematic review, six of which reported orthostatic intolerance symptom prevalence.

### 3.2 Participant and study characteristics

Table 1 summarises the study and participant characteristics. Overall, 3446 participants were included (community-dwelling older adults (n = 1598) [23,24], geriatric outpatients (n = 1465) [2,3,[17], [18], [19],[25], [26], [27]], institutionalised (n = 116) [28] and a mixed population (n = 267) [29]) with a mean age of 75 (6 SD) years (56.5% females). The number of participants asked to report symptoms was 968 with a mean age of 73.4 (6.1 SD) years, 56% being female.

**Table 1:** Study characteristics of included articles, stratified by initial orthostatic hypotension definition.

First author, year	N	Valid measure-ments, N (%)	Age, mean (SD) years	Female, N (%)	Population/setting	Priori selection criteria	Study design	Rest (min)	Stand/sit (min)	Postural change	BP measurement (Technique)
**IOH within 15s**											
De Bruine, 2017 [25]	24	24 (100)	79.3 (7.7)	14 (58.3)	GO	COH	C	5	3	AS	Cont (PPG)
De Bruine, 2019 [26]	62	58 (93.5)	80.6 (7.0)	33 (56.9)	GO	–	C	5	3	PS, AS	Cont (PPG)
Hayakawa, 2015 [3]	216	199 (92.1)	Ctrl: 69 (7.1) MCI: 74 (6.8)	108 (50)	Memory outpatients	MCI	L	10	3	AS	Cont (PPG)
McDonald, 2017 [23]	353	297 (84.1)	73 (6.8)	40 (50.6)	General practice	–	L	10	3	AS	Cont (PPG)
Mol, 2018 [27]	109	109 (100)	81.7 (7.0)	60 (55)	GO	–	C	5	3	AS	Cont & Int (PPG & Sphyg)
Saedon, 2016 [29]	267	206 (77.2)	74 (6.6)	184 (68.9)	ED, GP, GO, hospitalized	Falls with (out) injury	C	10	3	AS	Cont (PPG)
Saedon, 2020 [24]	1245	1245 (100)	67.9 (6.9)	693 (55.7)	Community	–	L	5	3	AS	Cont (PPG)
**IOH within 15s + symptoms**											
Breeuwsma, 2017 [19]	127	104 (81.9)	68.8 (8.5)	45 (43)	GO∗	≥1 CVD, DM or HTN	C	5	3	AS, SS	Cont (PPG)
Romero-Ortuno, 2011 [17]	608	442 (72.7)	72.1 (7.1)	317 (71.7)	GO	–	C	10	3	AS	Cont (PPG)
**IOH within 15** **s, correcting within 30-60** **s**											
McJunkin, 2015 [2]	115	115 (100)	71.1	57 (49.5)	GO	–	C	5–10	1–3	AS	Int (Sphyg)
**IOH within 30s**											
Shaw, 2019 [28]	116	116 (100)	84.2 (0.9)	65 (56)	Aged care residents	–	L	15	15	PSS	Cont (PPG)
**OH within 1** **min**											
Bengtsson-Lindberg, 2015 [18]	204	204 (100)	76 (6.5)	128 (62.7)	Memory clinic	Dementia	C	10	10	AS	Int (Sphyg)

BP: blood pressure. C: cross-sectional. L: longitudinal. GO: Geriatric outpatients. Ctrl: control. MCI: Mild cognitive impairment. IOH: initial orthostatic hypotension. COH: classical orthostatic hypotension. ED: Emergency Department. GP: general practitioner. CVD: cardiovascular disease. DM: diabetes mellitus. HTN: hypertension. AS: active stand. SS: supine-sit. PS: passive stand. PSS: passive supine-sit. Int: intermittent. Cont: continuous. PPG: photoplethysmography. Sphyg: sphygmomanometer. GO∗: Geriatric outpatients with one or more of: diabetes mellitus, hypertension or cardiovascular disease.

### 3.3 Definitions of IOH, orthostatic intolerance and methodology

Five different IOH definitions were reported between the 12 included articles: IOH within 15 s [3,[23], [24], [25], [26], [27],^29^], IOH within 15 s with the presence of symptoms [17,19], IOH within 15 s correcting within 30 and 60 s [2], IOH within 30 s [28] and OH within 1 min [18]. The duration of the resting period spent supine prior to a postural change was 5 min [19,[24], [25], [26], [27]], between five and 10 min [2], 10 min [3,17,18,23,29], and 15 min [28]. Postural changes included were active stand [2,3,17,18,[23], [24], [25],^27^,^29^], passive supine-sit [28], both active stand or supine-sit [19] and a combination of both passive stand followed by active stand [26]. The period of time spent upright, either standing or sitting was between one to 3 min [2], 3 min [3,17,19,[23], [24], [25], [26], [27],^29^], 10 min [18] or 15 min [28]. Blood pressure was measured continuously with digital photoplethysmography [3,17,19,[23], [24], [25], [26], [27], [28], [29]], or intermittently via a digital sphygmomanometer [18,27] or wall mounted sphygmomanometer [2] (Table 1). Participants were asked to abstain from caffeine [29] and nicotine [23] from the night before and to have a light breakfast [23] or fast for 10 h [24] on the day of the measurement. Three articles stated the time of day in which blood pressure was measured, which was performed in the morning [3], 9am-1pm [23] or 9am-5pm [17].

### 3.4 IOH, COH and orthostatic intolerance prevalence

**Fig. 2:**
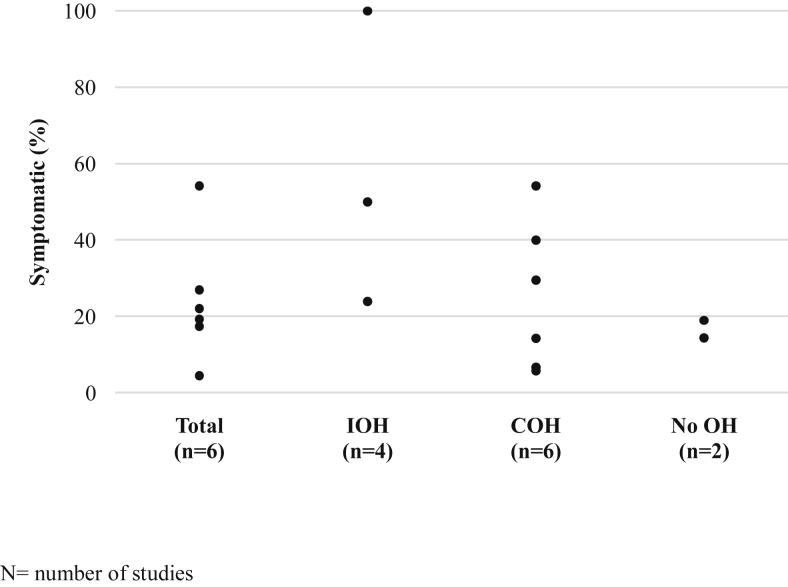
Prevalence of orthostatic intolerance symptoms reported in total populations (Total), populations with initial orthostatic hypotension (IOH), classical orthostatic hypotension (COH) and no orthostatic hypotension (No OH).

The prevalence of IOH ranged between 3.5 and 62.5%, and COH between 6 and 100%, as presented in Table 2. Overall, the total prevalence of orthostatic intolerance symptoms not specific to any orthostatic hypotension diagnosis ranged from 5 to 54% between the studies reporting symptoms (Fig. 2). Orthostatic intolerance symptom prevalence in participants with IOH was reported in four articles. The prevalence of orthostatic intolerance symptoms in participants with IOH was 24% [18], 50% [2] and 100% [17,19], dependent on the IOH definition used (Fig. 2). Two articles reported orthostatic intolerance symptoms independent of an IOH or COH diagnosis, with the prevalence being 4.5% [23] and 54.2% [25]. Two of the six articles also reported the prevalence of orthostatic intolerance symptoms in participants who had no IOH or COH diagnoses, the prevalence of orthostatic intolerance symptoms in these participants was 14.4% and 19% (Fig. 2). Symptoms most commonly reported included dizziness, light-headedness, blurred vision and instability [2,[17], [18], [19],^25^].

**Table 2:** Prevalence of initial orthostatic hypotension, classic orthostatic hypotension and orthostatic intolerance symptoms, stratified by initial orthostatic hypotension definition.

First author, year	N	OH (%)		Symptoms (%)				COH def.
		IOH	COH	Total	IOH	COH	No OH	
**IOH within 15s**								
De Bruine, 2017 [25]	Total, n = 24	54.2	100	–	–	–	–	COH ex. First 15s
	Normal, n = 24	–	100	54.2	–	54.2	–	
	Slow, n = 24	–	100	41.7	–	41.7	–	
	Fast, n = 24	–	100	75	–	75	–	
De Bruine, 2019 [26]	Total, n = 58	41.1	84.5	–	–	–	–	COH ex. First 15s
Hayakawa, 2015 [3]	Total, n = 199	62.5	6^b^	–	–	–	–	COH
McDonald, 2017 [23]	Total, n = 297	16.2	81	4.5	–	–	–	COH
	Falls, n = 30	–	82	17	–	–	–	
	No falls, n = 49	–	87	14	–	–	–	
Mol, 2018 [27]	Total, n = 109	29.4	76.1	–	–	–	–	COH
Saedon, 2016 [29]	Total, n = 267	16	54	–	–	–	–	COH
Saedon, 2020 [24]	Total, n = 1245	24.9	62	–	–	–	–	COH, ex. IOH within 15s
**IOH within 15s + symptoms**								
Breeuwsma, 2017 [19]	Sitting, n = 104	5.8	67.3	12.5	100	6.7^a^	–	COH ex. First 15s
	Standing, n = 104	16.3	66.3	22.1	100	5.8^a^	–	
Romero-Ortuno, 2011 [17]	Total, n = 442	19.2	94.1	19.3	100	29.5	–	COH
	Cluster 1, n = 112	3.6	80.4	17.9	100	14.3^a^	–	
	Cluster 2, n = 238	17.4	98.3	27.5	100	10.1^a^	–	
	Cluster 3, n = 92	43.5	100	44.6	100	1.2^a^	–	
**IOH within 15** **s, correcting within 30-60** **s**								
McJunkin, 2015 [2]	Total, n = 115	3.5	12.2	17.4	50	14.3	14.4	COH, sust.
**IOH within 30** **s**								
Shaw, 2019 [28]	Total, n = 116	11.5	44	–	–	–	–	COH
**OH within 1** **min**								
Bengtsson-Lindberg, 2015 [18]	Total, n = 204	10.9	34	27	24	40	19	COH
	Control, n = 50	0	13	2	–	–	2.3	
	DLB, n = 50	34	70	40	–	–	20	
	AD, n = 50	4	33	28	–	–	29	
	Advasc, n = 54	6	45	37	–	–	33.3	

OH: orthostatic hypotension. IOH: initial orthostatic hypotension. COH: classical orthostatic hypotension. COH def.: classical orthostatic hypotension definition. DLB: Dementia with Lewy bodies. AD: Alzheimer's disease. ADvasc: Alzheimer's disease with vascular components.

a Symptoms experienced not specific to COH diagnosis or no OH diagnosis.

b Systolic blood pressure range of deficit >30% from baseline at 60 s, (%). COH ex. First 15s: COH excluding the first 15 s after postural change. COH sust.: COH with sustained low blood pressure. COH, ex. IOH within 15s: COH without IOH occurring in the first 15 s post postural change.

### 3.5 Quality assessment

Eight articles were of high quality and four articles were of moderate quality (Supplementary Material 3). Of the six articles that reported orthostatic intolerance symptoms, three were of high quality [17,19,23] and three articles were of moderate quality [2,18,25].

## 4 Discussion

Orthostatic intolerance symptom prevalence ranged between 24% and 100% in older adults with IOH, which was dependent on the definition of IOH used. Literature on orthostatic intolerance symptoms in older adults with IOH is very limited and a variety of IOH definitions are being used.

### 4.1 IOH definitions and orthostatic intolerance symptom prevalence

Orthostatic intolerance symptoms are considerably prevalent in older adults with IOH and the variation in orthostatic intolerance symptom prevalence can be attributed to differences in the IOH definitions used. Definitions of IOH including symptoms as a requirement for an IOH diagnosis, such as ‘IOH within 15 s, with symptoms’ [17,19], exclude older adults with a large drop in blood pressure that are asymptomatic from an IOH diagnosis. This may be problematic as the majority of older adults with a large blood pressure drop upon standing have been described as asymptomatic [2,18,30]. The experience of orthostatic intolerance symptoms may rely on supine blood pressure and cerebral perfusion [31], the magnitude or rate of the blood pressure drop [32] and the capacity to maintain cerebral perfusion [33] while experiencing a drop in blood pressure upon change in posture. Individuals who experience supine hypertension may meet orthostatic hypotension criteria but still have increased or normal blood pressure and cerebral perfusion while upright [1,34,35]. To accommodate for this, an alternate cut-off of a drop in SBP of at least 30  mmHg occurring within 3 min of a change in posture has been proposed to diagnose COH in individuals with supine hypertension [1].

### 4.2 Clinical relevance

COH has been associated with poor clinical outcomes in older adults including a higher risk of falls [36,37], frailty [38], decreased cognition and physical function [39,40] and quality of life [12], coronary heart disease, heart failure, stroke and mortality [41] independently of the experience of symptoms. The presence of symptoms of orthostatic intolerance typically prompts clinical investigation and blood pressure monitoring [42], leading to lack of diagnosis and prolonged disease burden in individuals who are asymptomatic [43]. However, diagnosis of IOH is typically limited by the availability of continuous blood pressure monitoring devices in a clinical setting or lack of training in the administration and interpretation of beat to beat blood pressure recordings [44]. The relevance of asking patients to report orthostatic intolerance symptoms has been highlighted by its association with a higher prevalence of frailty, pre-frailty and functional decline in older adults with both IOH and COH [43,45]. However, not all individuals with IOH experience orthostatic intolerance symptoms and it is currently unknown if those who are asymptomatic may also be at risk of adverse clinical outcomes. Asymptomatic COH participants have been shown to have similar decreases in SBP as symptomatic COH participants [46], however, symptomatic COH in comparison to asymptomatic COH has been associated with a higher prevalence of adverse clinical outcomes such as syncope, falls and fractures [13,14,47,48]. Of 465 participants undergoing continuous blood pressure measurement, only 14 were diagnosed with COH, however, 164 participants reported orthostatic intolerance symptoms without being diagnosed with COH [42]. This implies that in line with the findings of the present review, the presence of orthostatic intolerance symptoms cannot indicate COH or IOH in older adults and it is essential to ensure the criteria of IOH or COH are met by measuring blood pressure for an accurate diagnosis. Therefore, measurements of blood pressure using an active stand test should be a part of routine care in older adults regardless of the prevalence of symptoms in order to screen for IOH in addition to COH [43,49], as management may prevent the potentially detrimental outcomes in older adults [12,37]. Blood pressure preferably should be monitored with continuous blood pressure devices, as intermittent measurements may not capture IOH accurately as the drop in blood pressure often occurs rapidly and before the cuff inflates.

### 4.3 Contributing factors to orthostatic intolerance symptoms

Many factors contribute to blood pressure fluctuations throughout the day, such as fluid intake, food consumption [50], time of the day [51], ambient temperature [52], medications [53], caffeine and nicotine [54] which may also exacerbate or diminish orthostatic intolerance symptoms [50]. Orthostatic intolerance symptoms in older adults are exacerbated in the morning and improve throughout the day, due to natural circadian fluctuations in blood pressure [55], fluid intake or medication [50]. Acute illness [56] and various comorbidities may cause the emergence or exacerbation of the drop in blood pressure experienced upon standing, which may also have an effect on the prevalence of orthostatic intolerance symptoms [57]. For example, hypertension [58], Parkinson's disease [35], renal failure [30] and associated anaemia [59] respectively can influence the prevalence of orthostatic intolerance symptoms and have been associated with COH, however the association with IOH is currently unknown. Longer periods of time spent supine before postural changes [21] and postural changes from supine to standing lead to a larger decrease in blood pressure than postural changes from supine to sitting [19] which can increase the likelihood for symptoms to prevail. For this reason, active stand tests are most reliable at detecting both IOH and COH compared with passive postural changes, as the initial blood pressure drop is not as pronounced or does not occur at all upon passive changes in posture [49,60].

### 4.4 Strengths and limitations

Only a small number of included articles reported the prevalence of orthostatic intolerance symptoms, despite reporting the prevalence of IOH diagnoses. Additionally, orthostatic intolerance symptoms experienced are asked independently of an IOH or COH diagnosis and therefore it is not clear if orthostatic intolerance symptom prevalence is associated to either IOH, COH or both forms of orthostatic hypotension. Heterogeneity between the populations of included articles may limit the generalizability of the results. A meta-analysis was unjustified as five different definitions of IOH were used between the six studies that reported symptoms.

## 5 Conclusion

A considerable proportion of older adults diagnosed with IOH report orthostatic intolerance symptoms, however, literature is limited. This highlights the need for further research on IOH and orthostatic intolerance symptoms, particularly in relation to whether older adults with IOH and orthostatic intolerance symptoms have worse clinically relevant outcomes compared to asymptomatic older adults with IOH.

## Author contribution

**Elena M. Christopoulos:** Formal analysis, Investigation, Writing - original draft, Visualization. **Jennifer Tran:** Investigation, Validation. **Sarah L. Hillebrand:** Investigation, Validation. **Peter W. Lange:** Conceptualization, Supervision. **Rebecca K. Iseli:** Supervision, Methodology, Writing - review & editing. **Carel G.M. Meskers:** Supervision, Writing - review & editing. **Andrea B. Maier:** Conceptualisation, Writing - review & editing, Methodology, Validation, Supervision, Project administration, Funding acquisition.

## Funding

This research was funded by an unrestricted grant of the 10.13039/501100001782University of Melbourne received by Professor Andrea B. Maier and the Medical Research Future Fund (MRFF) provided by the Melbourne Academic Centre for Health (MACH).

## Declaration of competing interest

None.
