# Supplementary material for: Initial orthostatic hypotension and orthostatic intolerance symptom prevalence in older adults: A systematic review
Source: Int J Cardiol Hypertens. 2020 Dec 8;8:100071. doi: 10.1016/j.ijchy.2020.100071 (PMC7803043; doi:10.1016/j.ijchy.2020.100071)
Supplement: Multimedia component 1 [file mmc1.docx]

**Supplementary Material 1.** Search strategy

**Database: Ovid MEDLINE(R) and Epub Ahead of Print, In-Process & Other Non-Indexed Citations and Daily <1946 to December 06, 2019>**

1     ((initial adj2 (hypotension adj3 postural)) or (postural adj3 blood adj2 pressure) or (orthostatic adj3 blood adj2 press*) or (orthostatic adj3 hypotens*) or orthostasis).ti,ab,tw. (6534)

2     (initial adj2 (orthosta* or postur*) adj3 (hypotensi* or (blood adj2 pressure))).ti,ab,tw. (39)

3     "initial BP drop".ti,ab,hw. (6)

4     1 or 2 or 3 (6540)

5     (aged or elder* or senior* or old* age or old* adult* or old* person* or old* people or old* men or old* women or geriatri*).ti,ab,hw. (5344037)

6     4 and 5 (3760)

**Database: EBM Reviews - Cochrane Central Register of Controlled Trials <November 2019>**

1.   ((initial adj2 (hypotension adj3 postural)) or (postural adj3 blood adj2 pressure) or (orthostatic adj3 blood adj2 press*) or (orthostatic adj3 hypotens*) or orthostasis).ti,ab,tw. (1221)

2.    (initial adj2 (orthosta* or postur*) adj3 (hypotensi* or (blood adj2 pressure))).ti,ab,tw. (8)

3.     "initial BP drop".ti,ab,hw. (2)

4.     1 or 2 or 3 (1224)

5.     (aged or elder* or senior* or old* age or old* adult* or old* person* or old* people or old* men or old* women or geriatri*).ti,ab,hw. (522387)

6.     4 and 5 (641)

**Database: Embase Classic+Embase <1947 to 2019 December 06>**

1.     ((initial adj2 (hypotension adj3 postural)) or (postural adj3 blood adj2 pressure) or (orthostatic adj3 blood adj2 press*) or (orthostatic adj3 hypotens*) or orthostasis).ti,ab,tw. (10436)

2.     (initial adj2 (orthosta* or postur*) adj3 (hypotensi* or (blood adj2 pressure))).ti,ab,tw. (68)

3.     "initial BP drop".ti,ab,hw. (6)

4.     1 or 2 or 3 (10443)

5.     (aged or elder* or senior* or old* age or old* adult* or old* person* or old* people or old* men or old* women or geriatri*).ti,ab,hw. (4700910)

6.     4 and 5 (3910)

| **Supplemental Material 2.** Newcastle-Ottawa Quality Assessment Scale (adapted for cross -sectional studies) |
| --- |
| A study can be given a maximum of one point for items 1, 2, 3, 4 and 7. A maximum of two points can be given for items 5 and 6. Maximum total score of 9 points. 0-3 = low quality, 4-6 = moderate quality, 7-9 = high quality |
| **Selection** (S): *Maximum 4 stars* |
| 1. Representativeness of the exposed cohort 2. True representative of exposed cohort aged 65 years and over with initial orthostatic hypotension * 3. Not representative or no description 4. Sample size: 5. Justified and satisfactory * 6. Not justified 7. Selection of the non-exposed cohort 8. From the same community as the exposed * 9. From a different source 10. No description of the derivation of the non-exposed cohort 11. Ascertainment of the exposure: how is initial orthostatic hypotension diagnosis made 12. Blood pressure measured continuously * 13. Blood pressure measured intermittently 14. No description or unclear |
| **Comparability** (C): *Maximum 2 stars* |
| 1. Comparability of cohorts adjusted for potential confounders 2. The study controls for age or sex * 3. The study controls for comorbidity, frailty or medication * 4. Cohorts are not comparable on the basis of the design or analysis controlled for confounders |
| **Outcome** (O): *Maximum 3 stars*   1. Assessment of IOH definition and symptoms: 2. Drop in SBP of ≥40mmHg and/or DBP of ≥20mmHg within 15 seconds with or without symptoms, recorded symptom prevalence ** 3. Drop in SBP of ≥40mmHg and/or DBP of ≥20mmHg within 15 seconds with or without symptoms, did not record symptom prevalence * 4. Other definition, recorded symptom prevalence * 5. Other definition, did not record symptom prevalence 6. No description or unclear |
| 1. Statistical test: 2. The statistical test used to analyse the data is clearly described and appropriate, and the measurement of the association is presented, including confidence intervals and the probability level (p value) * 3. The statistical test is not appropriate, not described or incomplete |

| **Supplementary Material 3.** Risk of bias quality assessment using the Newcastle-Ottawa Scale. | | | | | | | |  |  |
| --- | --- | --- | --- | --- | --- | --- | --- | --- | --- |
| **First Author, Year of publication** | **Selection** |  |  |  | **Comparability** | **Outcome** |  | **Score** | **Quality** |
|  | Representa-tiveness of exposed cohort | Sample size | Selection of non-exposed cohort | Ascertainment of exposure: continuous BP | Adjustment for potential confounders | Assessment of outcome | Statistical Test |  |  |
| Bengtsson-Lindberg, 2015 | * | * | * | - | * | * | * | 6 | Moderate |
| Breeuwsma, 2017 | * | * | * | * | - | ** | * | 7 | High |
| De Bruine, 2017 | * | * | * | * | - | * | * | 6 | Moderate |
| De Bruine, 2019 | * | * | * | * | * | * | * | 7 | High |
| Hayakawa, 2015 | * | * | * | * | ** | * | * | 8 | High |
| McDonald, 2017 | * | * | * | * | * | * | * | 7 | High |
| McJunkin, 2015 | * | * | * | - | - | * | * | 5 | Moderate |
| Mol, 2018 | * | * | * | * | * | * | * | 7 | High |
| Romero-Ortuno, 2011 | * | * | * | * | - | ** | * | 7 | High |
| Saedon, 2016 | * | * | * | * | ** | * | * | 8 | High |
| Saedon, 2020 | * | * | * | * | ** | * | * | 8 | High |
| Shaw, 2019 | * | * | * | * | - | - | * | 5 | Moderate |
| *Attributed point. Score 0-3: low quality, 4-6: moderate quality, 7-9: high quality. | | | | | | | | | |
